# A study protocol for applying the co-creating knowledge translation framework to a population health study

**DOI:** 10.1186/1748-5908-8-98

**Published:** 2013-08-29

**Authors:** Kathryn Powell, Alison Kitson, Elizabeth Hoon, Jonathan Newbury, Anne Wilson, Justin Beilby

**Affiliations:** 1 School of Population Health, The University of Adelaide, Adelaide 5005, Australia; 2 School of Nursing, The University of Adelaide, Adelaide 5005, Australia; 3 School of Population Health, The University of Adelaide, c/ PO Box 3200, Port Lincoln, South Australia 5606, Australia; 4 School of Medicine, The Flinders University of South Australia, South Australia, Australia; 5 School of Medicine, University of New South Wales, New South Wales, Australia; 6 The University of Adelaide, Adelaide 5005, Australia

**Keywords:** Knowledge translation, Population health, Knowledge creation, Knowledge translation model, Action research, Knowledge framework, Community health

## Abstract

**Background:**

Population health research can generate significant outcomes for communities, while Knowledge Translation (KT) aims to expressly maximize the outcomes of knowledge producing activity. Yet the two approaches are seldom explicitly combined as part of the research process. A population health study in Port Lincoln, South Australia offered the opportunity to develop and apply the co-KT Framework to the entire research process. This is a new framework to facilitate knowledge formation collaboratively between researchers and communities throughout a research to intervention implementation process.

**Design:**

This study employs a five step framework (the co-KT Framework) that is formulated from engaged scholarship and action research principles. By following the steps a knowledge base will be cumulatively co-created with the study population that is useful to the research aims. Step 1 is the initiating of contact between the researcher and the study contexts, and the framing of the research issue, achieved through a systematic data collection tool. Step 2 refines the research issue and the knowledge base by building into it context specific details and conducting knowledge exchange events. Step 3 involves interpreting and analysing the knowledge base, and integrating evidence to inform intervention development. In Step 4 the intervention will be piloted and evaluated. Step 5 is the completion of the research process where outcomes for improvement will be instituted as regular practice with the facilitation of the community.

In summary, the model uses an iterative knowledge construction mechanism that is complemented by external evidence to design interventions to address health priorities within the community.

**Discussion:**

This is a systematic approach that operationalises the translational cycle using a framework for KT practice. It begins with the local context as its foundation for knowledge creation and ends with the development of contextually applicable interventions. It will be of interest to those involved in KT research, participatory action research, population health research and health care systems studies. The co-KT Framework is a method for embedding the principles of KT into all stages of a community-based research process, in which research questions are framed by emergent data from each previous stage.

## Background

Population health is concerned with health outcomes within an aggregated group of people, and research on population health considers the impact of environmental and system variables on the distribution of those health outcomes [1]. It is a research domain that has received limited attention within the KT field. Two aspects have been identified as being relatively absent in population health studies: non-quantitative studies and the incorporation of people as agencies within a health system [2]. This protocol describes how the co-KT Framework incorporates KT into a population health research study.

The impact of KT on population health studies is of interest to the research community, as there is a need for empirical studies of the effectiveness of KT in improving the health of the population by changing practices [3]. Others have identified priorities for KT development work such as the need for better structural conceptualising of KT and the need to adapt KT strategies to context [3]. Further it has identified that the majority of researcher-practice frameworks address only one phase (sometimes two) of the process of research-practice translation [4].Within this study, we apply the co-KT Framework to that phase of KT concerned with moving discoveries into communities, and then extended to organisations and policy developers, which has been described as T3 [5]. This area is still being defined in scope [6].

Population health has been the subject of limited KT research approached in a systematic way [7], and there are very few KT intervention studies with a focus on public health and prevention and whole systems redesign [8].Lightfoot et al. (2008) describe ways of conducting population based health research in rural areas, emphasising early stakeholder involvement if the study is proposed by external researchers and suggesting a community needs assessment to help to build community and funding support for the proposed study [9]. Ogilvie et al. discuss a translational framework for public health research, shifting the focus to the improvement of population health through individual and collective determinants of health [10]. This is a clear difference from embedding evidence-based interventions. In public health planning frameworks generally, tacit knowledge is not referred to as a legitimate source of knowledge nor do they pragmatically suggest how this knowledge might be elicited [11].

This protocol describes how KT is being conducted within The Physiology of Health Systems: Port Lincoln as a Case Study (the LINKIN Health Study, National Health and Research Council project grant 627240, 2010–12). The research team is finding a way to link evidence to issues within a particular context rather than applying evidence and identifying barriers. The LINKIN Health Study is a mixed methods study of the population of Port Lincoln, South Australia (population: 14,000 people) [12]. The approach described in this paper is intended to provide detailed insight into the use of both formal and informal sources of health-care and into the extent and pattern of any mis-alignment between established morbidity and use or provision of services. This is in contrast to solely using epidemiological tools to map person-based data on use of health services and limiting the evidence base to information collected by quantitative methods.

There is no favoured operational KT approach for a total research project process. We found that KT frameworks tended to separate the knowledge generation process from the actual implementation of knowledge [13-15]such as those that addressed evidence-based practice or the diffusion of knowledge process, the effectiveness of KT implementation, in the form of interventions, or the uptake of evidence and articulating conditions for successful KT [16-22]. The KT framework we have applied (the co-KT Framework) does not begin by applying evidence-based practice but concentrates on allowing the emergence of context specific data.

Our framework is presented as a means whereby KT within a research study may provide a structured means of developing a shared understanding of a problem and its underlying factors [23].

Recognition of the move to develop community interventions that take account of the complex array of causal factors underlying health inequalities has been important to the study team [24]. We also wanted to shape a methodological approach that was not confined to a single-issue intervention but rather used the research process to open up the possibility of addressing community systemic conditions [24]. The framework goes some way to working on the little addressed aspect of the ways in which the interaction between multiple players in a system might be managed and knowledge intermediation (managed processes of knowledge interaction) [25,26].

This protocol describes how a theory informed conceptual framework for KT will be applied in a population health study that sets out to formalise collaborative approaches within the design, whilst differentiating between the roles of both the stakeholders and the researchers. The protocol describes the application of a KT framework to a mixed methods population health study. A separate protocol relates to the mixed methods design itself. The protocol demonstrates the deliberate incorporation of the theories of engaged scholarship [27,28] and practice of participatory action [29] as we put research generated knowledge as a key driver of improved health care. In particular, we see these underlying theoretical foundations as enabling the co-learning and co-construction of knowledge and understanding of that knowledge, the acknowledgement of a range of social and environmental factors that impact on health, and including community involvement at all stages of the research process [29]. Action research and engaged scholarship explicitly promote the concerns of the local context and have the capacity to generate knowledge that addresses local concerns [30]. There is an assumption within these two theories that there may be multiple sources of knowledge and that people within the community possess valid knowledge. We do not suggest knowledge is objective nor a set of context-free facts that must be packaged for communities but rather we are responding to the practical recognition that knowledge translation and intermediation can be sites of conflict [25,26].We view knowledge as a research tool and point of common engagement; knowledge is to be constructed and shaped, in our study, for the purpose of improved population health.

Action research and engaged scholarship both focus on the researcher– community relationship as a combination that will yield effective research rewards [30]. We anticipated that this theoretical base would facilitate the framing of research questions that were more meaningful to the community (for example, rather than specifying a reduction of ill-advised rates of health incidence, we could frame our issues for address in terms of what might most benefit the community). There has been recognition of the interface between participatory oriented research and KT and the co-construction of knowledge in a relationship that includes stakeholders and researchers [31]. Community-based participatory research methods have directly addressed the researcher and community relationship, and its role in the community intervention process [24]. Clavier et al. found that strategic translation supports the research process and facilitates the ongoing collaborative involvement of stakeholders [31]. A further point of congruence between KT and participatory action research is that community-based participatory research places significance on actionable knowledge, and knowledge produced through relationships and collaborative practice [29]. It also considers the problem of synthesising evidence which is important in a mixed methods design.

## Methods

The LINKIN Health Study examines the health system functioning within the rural population of a South Australian town and takes place at the nexus of university and community research. It aims to build knowledge for the purpose of health reform between these two contexts, using quantitative and qualitative data collection as a foundation on which to develop suitable health interventions [12]. The co-KT Framework is to form the integrating and synthesising thread through the research process and is led within the research team by a nominated sub-group which is a key feature of the study protocol.

### Aims of the co-KT framework

Within this population health study, a KT mechanism was developed to:

• Produce community-based knowledge for application within and by the community to increase the effectiveness of health service delivery and health outcomes for agreed priority groups, and in an integrative, cross-disciplinary way.

• Allow for the community-based adaptation of externally derived knowledge to local health issues guided by the research team.

### The study context (or research setting)

Port Lincoln is a major city on the Eyre Peninsula, South Australia. Port Lincoln is one of the nation's biggest combined agricultural and fishing centre. It has a population of 14,726 (Australian Bureau of Statistics Estimated Resident Population 2010). In the surrounding region of the Eyre Peninsula the population has increased by 18% between 2001 and 2011 [32] and demographic projections state that the population of Eyre Peninsula will have more people aged between 55 and 74 years over the next decade or so [33]. Within Port Lincoln’s population, Aboriginal people make up 5.5% and overseas born people comprise 8.9% with half being born in north-west Europe. In terms of the physicality of their occupations, 18% classify themselves as labourers and a further 15% have a trade or technical occupation (ABS ERP 2010). Prior to the study commencing we were aware that on Eyre Peninsula avoidable hospital admissions are higher than South Australia and Australia (for diabetes complications, asthma, chronic obstructive pulmonary disease, ear/nose/throat infections, dehydration, gastroenteritis), and 71% of all deaths in Eyre Peninsula at ages 0–74 years in the period 1997–2001 were considered to be avoidable (cardiovascular and cancer) [33].

### Evidentiary inputs

The key evidentiary inputs for this protocol are:

• A purpose designed health census [12]

• Subsequent surveys of those persons reporting specific prevalent conditions in the foregoing health census and consenting to be re-contacted

• Focus groups with service providers.

### Community engagement

Key community engagement information sources are:

• Face to face discussions with local service providers

• Face to face discussions with the residents (that is, the potential service user population).

• Engagement of policy administration, and regional governance bodies.

### Establishing linkages with the community

An important part of the study design is to establish connector mechanisms between the researcher context and the study context. The value of knowledge brokers, boundary spanners, networks and communities of practice, and building in exchange processes between researchers and knowledge users during a research study has been highlighted [23]. These are mechanisms for researchers to connect with the community [34]. In our study, boundary spanners and other connectors are ways of drawing on tacit knowledge within a community. We are also including within our sphere of engagement stakeholders who may influence community change at regional levels such as local government and organisational representation.

The study design incorporates the use of a variety of other tools for establishing community linkages such as internet based tools to mitigate against inability or lack of incentive to use person based opportunities (including a purpose developed web page and facebook page), publicly available media (local newspaper and national/state newspaper, radio), purpose developed study related information brochures and flyers, holding public meetings, speaking at organisation meetings including regional bodies, and promoting direct contact details (a project email and contact telephone numbers).

### Basis of co-KT framework

**Figure 1:**
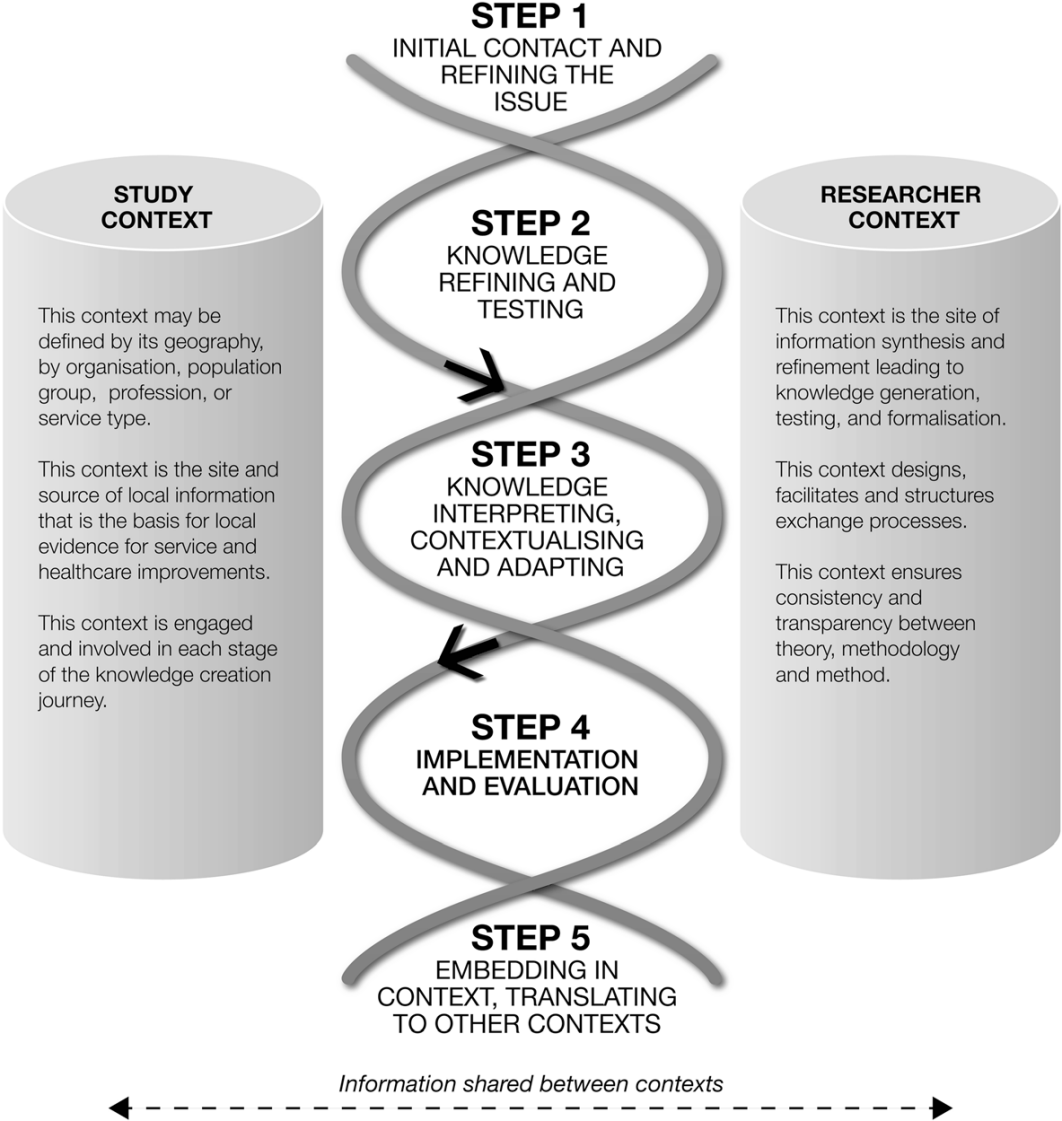
Information to knowledge in the co-creating KT (co-KT) knowledge translation framework.

In our model (shown diagrammatically in Figure 1) the researcher context and the study context form the pillars between which knowledge creation and its exchange is bi-directional. The knowledge creation process is guided by synthesising outputs from the researcher and study contexts. This process may be described as dialogic, and iterative, across the researchers and the knowledge users [35]. The framework provides for a theory based design of combining context-derived information, explicitly acknowledging the value of tacit knowledge that is addressed in combination with researcher understandings of evidence-based options. The co-KT Framework [36] requires the input and collaboration of the community and draws on action research, including Participatory Action Research (PAR), and engaged scholarship [27]. The framework is not prescriptive about the type of data that forms the evidence base.

The steps are designed to construct a co-learning experience about issues raised through the community and framed as health related problems. Intelligence is shared and reflected upon: a process of ‘curating knowledge’ (turning information into knowledge and identifying the connections between it and the research questions; making tacit knowledge explicit; integrating knowledge and shaping it so it is usable [37]). To illustrate the protocol, Table 1 is a ‘walk through’ of what is involved in undertaking each step. Within each step there will be knowledge particular to each group of stakeholders, for which certain tools or methods will be selected, and this will be undertaken in agreed ways by the research team (strategies).

**Table 1:** Co creating knowledge translation method

**Co-create KT Step**	**Knowledge sought**	**Tool(s)**	**Strategies**
**Step 1: Initial contact and framing the issue.**			
**Contact between the study context and research context occurs in response to a broadly phrased issue.**	**Information from the study context that covers a broad spectrum within the issue(s).**	**Data gathering tool(s) that will generate a pool of information from which subsequent inquiries can be refined.**	**View research as a means and not an end.**
			**Establish a KT research lead and advisory team within the study.**
			**Identify persons in both contexts as points of contact and information.**
**LINKIN EXAMPLE**			
**Issue: What is the health status of the people in Port Lincoln and how do they utilise health services?**	Researcher context made initial inquiry of population-wide incidence of conditions.	Quantitative data tool:	Appointment of 3 boundary spanners.
		Health Census – a written structured survey to population of study context via households.	
			Inclusion of local people as part of the Health Census operational delivery team.
			Use of varied media to convey information about the research Create a presence and identity by participation in local public events.
**Step 2: Refining and testing**			
**Research team lead the knowledge refinement process (of data and local evidence into context-relevant knowledge) by obtaining the perspectives of multiple stakeholders.**	**Contextual information to interpret the quantitative data.** **Qualitative data on defined aspects of the initial issue.**	**Community engagement strategies, participatory action research, information and knowledge products, communication strategies.**	**Use of an action research approach to methodically explore a problem within a designated context.**
			**Use of facilitators, connectors, boundary spanners, knowledge brokers.**
**LINKIN EXAMPLE** **Selection of four key issues such as specific health conditions and health service user groups in Port Lincoln.** **Comparison of information gathered with other data sources such as national surveys.**	Validation and explanation from the study context of the Health Census results. Knowledge that was related to the condition types nominated for further research. Knowledge on equity of experience within the study context. Knowledge on social determinants. Computer Assisted Telephone Interviewing (CATI) results to be communicated to stakeholders and provide opportunity for input.	Production of recorded source data into accessible forms for the community (newsletters, project website, local radio, local newspaper, printed copies of data presentations). Consultation strategy that included identified stakeholders (health service providers, residents, and key organisations). CATI (telephone) survey to ‘Health Census recontactees’ to obtain more condition specific information from study context.	Boundary spanners. Large scale campaign Community meetings Opportunities for open discussion (eg library). Focus groups. Creation of knowledge products (newsletters, webpages, hard and electronic copies of data presentations). Use of varied media channels (radio, newspaper, internet).
**Step 3: Interpreting, contextualising and adapting**			
**Local evidence is refined and tested against the existing evidence. Contextual information is incorporated into the evidence base to provide a basis for adapting the knowledge to form the basis for intervention ‘prototypes’ to be introduced and tested in the study context.**	**Customising intervention for practitioners involved.**	**Methods of developing and/or canvassing options with those stakeholders affected.**	**Feedback to study context of interpretation of evidence base.**
			**Development of options to address the issues.**
	**Agreement on interpretation of implications of knowledge base. Identification and prioritisation of key aspects to address.**		
			**Part of the process of making knowledge useful: interpretation, negotiation, debate. The knowledge needs to be linked or related to what is already known or experienced within the community.**
		**Audit and feedback mechanism to providers participating in the intervention development.**	
		**Participant observation, Questionnaires, Interviews, focus groups.**	
**LINKIN EXAMPLE**	What stakeholders think of current recommended best practice.	Questionnaires	Reference Bone and Joint literature review.
**For the Bone and Joint condition group**			
	Knowledge used to select features that will be addressed through pilot interventions.	Interviews, focus groups.	
**This step would involve the development of an intervention(s) that takes up community-based knowledge and is includes shaping by agents and participators within the context. The LINKIN study has not defined its interventions as yet.**			
	Dialogue with stakeholders during the development of the intervention.		
	Perceived impact of intervention by study context.		
**Step 4: Implementing and evaluating**			
**Involvement, trial uptake and response to interventions.**	**Evaluation data.**	**Communicate results and outcome of evaluation.**	**Consultation and evaluation strategies.**
	**Extent and effectiveness of intervention uptake and implementation.**		
			**Use of knowledge utilisation strategies.**
		**Use of knowledge utilisation measurement tools.**	
			**Use outcome measures for each level of the health system: patient level, health practitioner level and system (or organisational) level.**
**Community engaged in evaluating the interventions and modifications for ongoing use.**			
	**Qualitative data on why an intervention was successful or not effective, and how it could be improved.**		
**LINKIN EXAMPLE**			
**An intervention will be evaluated in real-time to monitor its reception and response in the community. This step is framed by examining how we would define and resource the intervention.**	Knowledge about the features of the intervention to retain in sustained interventions.	Routinely collected data (such as from audits).	Use of knowledge broker role.
	Context appropriate responses to evaluation data and extent of agreement with evaluation data.		
		Semi-structured discussion groups.	

**An example could be how professionals might work better to facilitate referral pathways that work within Port Lincoln.**	Perceived impact and sustainability of intervention by study context.		
			Establish an awareness of feedback being elicited at completion of evaluation.
**Step 5: Embedding into context and translating to other contexts**			
**Within the research context, evidence is formalised for local community and for the wider scientific community.**	**Knowledge that is to be included in final and lasting knowledge products.**	**Guidelines**	**Communication strategies of research outcomes and ongoing plans.**
**LINKIN EXAMPLE**			
**Following the intervention the research team leads consideration of how it might be sustained and in what form.**	How might this influence funding packages and reform taking place in primary care?	Discussion groups with key agents and participators from context.	Inform the national health agenda
		Use of guidelines and process documents.	
	Elements of the intervention that are particular to this context and how adaptable the intervention is to other contexts.		
**How does it lead to new research questions?**			

#### Step 1: Initial contact and framing the issue

In step 1, the key activities are to begin a relationship with the study context and commence data collection. Information, using both an initial data collection tool (a health census) and community relevant data (profile of health services both locally available and available from external providers on a regular scheduled basis) will be collected that will be used as a basis to build a shared understanding, which is cognisant of the community’s experience. Exploration of the prevalent health conditions is to be undertaken, taking into account the community’s perspectives and health care experiences and the information needed to respond to the research questions.

Key contact people within the study context will be identified to provide information about the study context that might help to shape the research approaches in subsequent phases of the study. In this initial step, we (the research context) will emphasise being open and transparent about sharing and collaborating with the study context. This is facilitated by the dissemination of research study descriptions (through hard copy and electronic information sources and promotion through information media).

#### Step 2: Refining and testing

In Step 2 the knowledge base will be refined and its level of detail increased for application to the development of interventions. The research team will steer an iterative process of conveying information between the study context and the research context, with contributions sought from both contexts in this incremental building process. This reinforces the intent to cultivate respected relationships as an investment in the future acceptance of interventions.

Issues for ongoing and in-depth research are defined and agreed by considering shared information (for example, through face to face contact).To do this, in preparation for the co-creation of knowledge, information gained to date will be prepared into products that may be discussed with the study context (powerpoint presentations, pamphlets, and summary documentation). The strategies for knowledge exchange and building include community engagement strategies, participatory action research methods, focus and discussion groups, information and knowledge products, and communication strategies. Of special value is the incorporation of community connectors such as boundary spanners, who are able to speak at community organisations and forums.

This process of data gathering, sharing and co-creating will take place over several weeks.

### Evaluation

Steps 2–5 include the collection of evaluation data for the purpose of testing the co-KT Framework. For example, feedback mechanisms (questionnaires) will be used for the variety of discussion forums that will include consultations with close to all health service providers in Port Lincoln as well as community support groups, focus groups for discipline specific providers, community group discussions and open residential discussions. This step is about commencing dialogue with the community and identifiable stakeholders (in roles distinct from soley community) and disseminating a first stage of data collection. It includes ascertaining key priorities. In addressing the importance of evaluation we have drawn on Buykx et al’s framework to monitor the impact of health services research [38]. Key sources of information for evaluation purposes will be the study website ‘hits’, the extent of coverage of stakeholders (percentage of all stakeholders with whom an initial dialogue was held), feedback data from questionnaires following consultations.

#### Step 3: Interpreting, contextualising and adapting

The goal of step 3 is to develop a body of evidence on which to target interventions and their subsequent piloting. This will be based on the collation, synthesis and analysis of knowledge from steps 1 and 2, and communications and discussions with the study context. The meaning of the knowledge should be collaboratively interpreted, for example, distinguishing between clinical practice issues and service gaps. Analysis of this evidence and guided discussions with the study context will support the determination of the principal issues to address, articulate underlying systemic props or confounders for better health care options.

Methods used in this step will include the use of knowledge products and targeted presentations and discussions with those stakeholders likely to be impacted by any planned change. Focus groups with relevant health service providers (relevance determined by involvement with the condition or health issue being addressed), and community groups are further methods of creating knowledge transfer and exchange opportunities.

### Evidence synthesis

One of the issues for complex research projects in which there are multiple sources of knowledge is the synthesis and assemblage of that knowledge. There are five steps in the co-KT Framework and knowledge is gathered in each of these steps with the intent of translating knowledge into practice. As a knowledge base is being cumulatively built upon from different sources, one of the problematic aspects can be synthesising and making sense of the information at hand. We have constructed an evidence synthesis matrix (shown in Table 2) that is a sequential way of working through information gathered in preceding steps to work towards the development of interventions. Essentially, Table 2 shows how qualitative and quantitative information are aligned with the goals of the research study to allow for decisions about interventions that will improve health outcomes for emergent community health issues based on evidence of suitable interventions. The arrow shows the direction of progression.

**Table 2:** Evidence synthesis matrix

**Steps**	** Knowledge bases to conduct co-KT**	**Content and data analysis**
	** Steps**	
**1**	**First data collection set**	Conditions seen to impact on the population and key associated characteristics
**2**	**Emergent consultation issues**	Informative issues raised by health services and residents
**3**	**Further data collection**	More in depth understanding of population use of service providers and key concerns relating to health conditions of interest
**4**	**Emergent consultation issues**	Informative issues raised by health services and residents
**5**	**Application of knowledge: intervention development**	Features of the health system to address:
		Distillation of a range of health system features based on context related knowledge base formed through preceding steps.
**6**	**Question: is there an intervention to...........[specify purpose]**	Agree on intent of potential intervention and explore wider evidence base and how interventions might be aligned with robust context related knowledge base.
**7**	**Define outcome expectations**	Nominate specific outcomes of changes introduced

### Evaluation

One aspect of interest in the evaluation will be on the quality of the data obtained in relation to its use for developing interventions that address emergent issues. Evaluation will also need to consider how satisfactory was the process for determining the emergent issues (recognising that not everyone is likely to agree with what has been selected from among several health issues as priorities to be addressed).There are two aspects of the study for which evaluation of this step will be especially informative: firstly, the amount and content of knowledge available to the researchers and secondly, how well the connections were able to be drawn between the data collected and priorities and health issues targeted for address in this study. To evaluate this component we propose to have a guided researcher reflection session in which the multi--disciplinary research team will respond to questions on these aspects from the researcher context perspective. From the study context, extent of concurrence as to priorities emergent from the data will be considered (responses to dissemination of data for example, suggestions of alternative priorities, expressions of disagreement via telephone calls, website/twitter/email posts, and records of meeting discussions on the data and priorities).

#### Step 4: Implementing and evaluating

The next part of the study protocol is to implement interventions, premised on contextually valid evidence, on a pilot basis. The interventions will have been determined through the synthesis process and canvassed with key stakeholders with their input in shaping the implementation process. The particular interventions are intended to be based on factors that will have the most impact on the priorities identified through engagement and interaction processes and from the evidence synthesis process.

Here the research group will be instrumental in continuing the knowledge gathering process of how well interventions have been implemented and the degree to which outcomes are being achieved. Project management responsibilities will be established within the researcher context and regular communication maintained between the researcher and study contexts. The study context is to be engaged and involved in implementing the interventions, and monitoring their usefulness. The stakeholders must be informed as to the monitoring and evaluation process and understand the mechanics of the interventions.

Suitable mechanisms for auditing (monitoring) the intervention pilot will be introduced. Written instructions about the intervention for implementation, including data collection expectations and templates/methods will be provided. Practical aspects include agreeing on the length of time for which monitoring will be undertaken and allocating responsibilities for data collection (this will be dependent on the nature of eh interventions determined).

During the implementation phase supports must be considered for those practitioners who face the most change due to interventions introduced.

### Evaluation

An important aspect in step 4 is the need for researchers and the study context to actively critically reflect on the suitability and effectiveness of the interventions for the desired outcomes, and ease, and cost-effectiveness of implementation. This should be undertaken after an agreed monitoring period and time for data collection. Exploratory measures will be collected during this phase to ensure usefulness for longer evaluation data collected to be established in Step 5. The information and implications will be shared, discussed and deliberated with reference to next steps in semi-structured discussion groups involving stakeholders. We highlight that our evaluation here is concerned with the co-KT framework application to which this protocol addresses, which must be distinguished from the broader research protocol which will assess the effectiveness of the interventions to improve health outcomes (in accordance with the overall research project aims).

Evaluation for this co-KT framework step 4 will be directed to how well the design of the intervention has translated into practice and is addressing the intended health issues prioirities agreed in step 2. Participant feedback (implementers of the intervention and recipients of the intervention) will be a key source as well as statistical data from the point of delivery (unable to be specified until intervention formulated). A further important feature to include is evaluation of population reach, that is, the extent to which the intervention reached those for whom it was intended.

#### Step 5: Embedding into context and translating to other contexts

Knowledge from the evaluation of Step 4 is to be assessed and considered by both the researcher and study contexts. Part of the study protocol and design of the co-KT Framework is to follow-through the pilot stage to put in place one or more health reforms to lead to better health outcomes that might be evaluated over a longer period. Alterations to the interventions may need to be introduced, and this is where context related engagement will impact the success and uptake of research outcomes. A major focus at this stage is ensuring stakeholders are engaged and committed to sustaining and owning the new practices that have emerged from the research study. A practical issue is the inevitable withdrawal of the research team from leading the process and the need to embed ongoing monitoring of the final determined intervention(s) for an agreed subsequent period.

Key tangible aspects to mark this part of the study protocol will be:

• a finished set of guidelines as to changes to be implemented as having arisen from the research study

• a communication strategy for research based products developed by the research team and made accessible to the study population

• study results will be communicated to the study context in the form of knowledge products developed to translate the research findings and face to face communications. It is also likely that the researcher context will produce other knowledge products such as written publications about outcomes, learnings and interventions for wider communication beyond Port Lincoln or in compliance with funding bodies.

### Evaluation

Evaluation of this step will take into account perceived utility of the interventions in relation to the context issues addressed (by users) and soundness of evidence base on which they were formulated (researchers). There will also be an evaluation of ease with which they able to be embedded within the health system and communication of what they were to improve within the population health system. It is intended to evaluate how well the stakeholders involved in the implementation of the interventions were engaged. These evaluations will take qualitative forms.

### Description of Co-KT Framework components and features

#### Knowledge

Knowledge is seen in the Co-KT Framework as the tangible currency that is created, used, translated and discussed between the research context, the study context and broader interested contexts (journal audiences, policy makers, other communities). Herein knowledge features as a valuable commodity that is formed through the collaborative efforts of the study and research contexts. It is information that has multiple sources (health service users, health service providers, and activity data, comparable community data, evidence based literature) and includes tacit community knowledge.

#### Knowledge products

Knowledge products are critical to the co-KT Framework. We have operated on the principal of disseminating all data in summary form to the whole community. The value and utility of the knowledge products is enhanced if a degree of deliberation is taken in preparing them according to intended usage and target section of the population. The LINKIN Health Study has found that knowledge products from an early stage have generated requests from the study context for knowledge products that are specific to organisational needs; for example study context specific information to provide supporting evidence for grant applications and to generate discussion on policy issues.

#### Evidence

We define evidence as information derived from empirical data, qualitative data, researcher observation, and comparable externally derived information (clearly identified as such). They are the basis for producing knowledge products.

#### Context

In the co-KT Framework, two contexts are differentiated. The study context is place-based and defined as the environment, place or circumstances targeted for situating the research. In other studies, the context might be an organisation, several organisations linked by treatment or patient pathway for example, or geographical population (such as a suburb) or population segment (such as males). The researcher context is defined as the person or group responsible for conducting research within and about the study context. The researcher context is bound by the research questions, ethics, timelines, a defined budget and expected outcomes for external stakeholders.

#### Facilitation

Facilitation (easement) has been emphasised as important in knowledge translation when it comes to the implementation of change. Kitson et al. (2008) propose that facilitation will be more effective if the context (of study) is understood and ‘diagnosed’, and interventions are tailor made to the context or circumstances of implementation [39]. Steps 1–3 of the co-KT Framework accommodate this function.

#### Use of connectors

The transference of knowledge between the two contexts (researcher and study) requires mechanisms to form bridges and connect the contexts. We see this as a two-way flow, to link university and research users [40]. People may be assigned roles as bridge builders in varying capacities, or as linkage personnel who are able move between the research context and the study context [5,41,42].

The purposes of such roles are to:

• Mediate between the two contexts and moderate, translate and explain knowledge

• Identify other people or resources to facilitate the study project

• Provide culturally specific information that allow for the appreciation of community traditions, symbolic behaviours or practices that may be relevant to improving population health outcomes [5].

The role of these connectors may be more or less formalised in knowledge translation; formal such as actively adapting information for audiences, or less formal in that they speak and interact across contexts, talking providing key messages in terms that are understood. Knowledge brokers are conduits between the research context and study context and decision-makers, transmitting information across contexts, and they may work as boundary spanners [41,42]. As a contrived device, they may also be considered a way of intervening within the pattern of knowledge conveyance in the context landscape [42], adopting a more direct, guided, rapid and deliberate method that is controlled largely by the research context. This role may also be used as a device to overcome dysfunctional communication or the absence of communication within the study context. Key connector roles we have identified are: an office holder in local government, a researcher prominent in the local Aboriginal community, and a local medical practitioner who is also a chief investigator in the LINKIN Health Study.

#### Stakeholder engagement

There is an acceptance that stakeholder participation in research is more likely to strengthen consensus for change and acceptance of models (because stakeholders feel their interests have been included in the model development) [43]. In the co-KT Framework, the term ‘stakeholder’ is understood to include knowledge users, health and service providers and representative groups. There is no distinction between public and private entities.

### Ethical issues

Ethics approval to conduct this study was received from the Human Research Ethics Committee, University of Adelaide.

## Discussion

The co-KT Framework is a theory based model for knowledge translation practice that has been shaped by the requirements of a particular population health study of a mixed methods design. We see the co-KT Framework as meeting a need faced by researchers, and in particular, multi-disciplinary teams of researchers when they strive to conduct a community-based study or population health study, to achieve research aims through the development of context- relevant knowledge. This resonates with the call from Hartling et al. (2007) who state that there is an urgent need for a better understanding of KT interventions from a multi-disciplinary perspective, KT interventions that are designed to target multiple professional groups, and that health care delivery improvements will come from interdisciplinary collaboration [44]. There is also a growing movement to reduce the distance between knowledge development and knowledge use, and linking this with the need to develop community interventions that take account of several sources of health inequalities [24]. To address such complexity, there must be opportunity to have an understanding of how each party to the research process (researchers, communities and stakeholders) frame a problem [45]. We believe we have opportunity for discursive discussion within the framework steps.

The co-KT Framework recognises the translation derived elements separated by Clavier et al. needed to operationalize a framework; incorporating cognitive practices (managing the research contents), strategic methods (to conduct the research process and manage relationships across stakeholders) and logistic practices (that relate to practical coordination) [31].

KT has been criticised for not considering sufficiently methods whereby the end-users of interventions or innovations might critically reflect on contextual related factors that might influence the embedding of an innovation within a context of practice [46].This protocol activates the engaged scholarship theoretical basis of the co-KT Framework in its participative form of research for obtaining the perspectives of different stakeholders and for recognising different sources of data to develop the most useful knowledge to address complex problems. Action research and PAR and engaged scholarship contain principles of enjoining and involving both academic researchers and situationally defined stakeholders within the dynamics of building knowledge around a framed issue. The protocol in particular illustrates how the expertise and knowledge of researchers and communities might be brought to bear on the health systems of a regional place [28]. The co-KT Framework incorporates the study context into the process of knowledge to evidence and evidence to innovation design, inviting critical reflection. Here, we demonstrate in this approach a view that community engagement for KT is more than smoothing a path to the implementation of interventions; it allows for an understanding of how public health policies and local context factors combine structuring the system, the reality, that must be navigated by individuals as the end-users to self-manage their health.

We appreciate that in community-based KT work there can be tension between what is considered evidence between clinicians and community-based organisations [47]. Kothari and Armstrong state that traditional KT approaches may not be suitable for all health service delivery systems [47]. Co-KT, as an iterative knowledge creation and translation process, may be better suited to community-based research. By incorporating KT at the knowledge creation (evidence building) stage, we have emphasised our focus on health system re-design and treatment of all stakeholders as part of a total health system serving a defined population.

An important aspect of the incorporation of KT within a population health study we suggest is the direction of community engagement into a form of social action that results in a health system change for the community that goes beyond simply research participation or engagement by a university [2]. How well this will be done remains to be seen but it is important at this stage of the protocol design that inclusion of the community in the knowledge building is more purposeful than local relationship building, and allows for the articulation by stakeholders of change options.

The protocol recognises the value of the social processes used to undertake our research [2]. We have emphasised face to face communication as a key form of relationship building and communication within our framework [29] and recognise that this poses its own dynamics and difficulties with organisation. However, we also consider this a better way to influence the credibility of the outcomes of our research [29].

We are aware that there is a shortage of rigorous operational studies or evaluation of KT strategies [8]. Dagenais et al. (2009) note that few studies present results on the effects of populations that are the subjects of interventions [3]. The co-KT Framework incorporates methods of evaluation within each step and at the completion of the research study and attention will be given to collating evaluation data from the LINKIN Health Study. We view the consulting itself as an interactive knowledge transfer tool and its effectiveness will be targeted for evaluation by qualitative means [48]. Methods of evaluation include feedback questionnaires, non-participant observation, interviews, assessment of the relevance of documentation, and intervention specific data. Simple knowledge and research utilisation questions will be employed as these have been demonstrated to be effective [49]. One of the dimensions intended for evaluation is ‘population reach’, that is, how many people did interventions resulting from the research impact upon?

### Challenges

There are likely to be several predictable challenges in implementing a framework of this scope we foresee pragmatic difficulties to be:

• Limited staff resources who will be concurrently engaged in information analysis and needing to prepare outcome based knowledge products to maintain momentum gained following data collection

• Engaging with busy health professionals at times that coincide with research steps and do not hold up the information gathering processes too long

• Cultivating local trust when seen to be inclusive of various stakeholders and building a ‘shared project culture’ [50]

• Achieving project timeframes when both community engagement and quantitative data analysis are features of the research design and can be time-consuming

• Avoiding ‘over consulting’ the community in fulfilling the need for both information collection and evaluation.

We also anticipate challenges where system reforms or interventions are perceived as impacting on accustomed ‘rewards’ systems or practices that are sustained within the present local context that benefit some and not others.

The findings of the current study will further implementation science by presenting a systematic method for a KT research process in a population health study that does not have an intervention as its starting point. It begins by eliciting the emergent predominant health issues from the local population. In contrast to other models, co-KT has provided the framework in which the knowledge user community, or study context, may be involved at the commencement of the knowledge building process [22]. Being able to report on its empirical use, both positive and unexpected outcomes will be of value to those researching health in the community and addressing the need for complex interventions.

## Competing interests

The authors declare they have no competing interests.

## Authors’ contributions

All the authors form the Knowledge Translation group within the LINKIN Health Study team. KP has led the writing and editing of this paper, literature review, prepared drafts, and detailed the method of the Co-KT framework. She is responsible for implementing the co-KT strategy within the LINKIN project. ALK led the conceptual development of the co-KT Framework and is responsible for the KT activity within the LINKIN project. She positioned its main content in relation to the conceptual paper describing the framework, revised drafts of the paper with KP and refined concepts. EH contributed to the design and implementation of co-KT strategies for this project, commented on drafts and is project lead for LINKIN. JN commented on drafts and is one of the local boundary spanners in Port Lincoln. AW has facilitated stakeholder meetings with KP and EH in Port Lincoln and has reviewed drafts. JB leads the conceptual and research design of the LINKIN health study and has reviewed all drafts. All authors read and approved the final manuscript.
